# Supplementary material for: Fluid–structure interaction simulations outperform computational fluid dynamics in the description of thoracic aorta haemodynamics and in the differentiation of progressive dilation in Marfan syndrome patients
Source: R Soc Open Sci. 2020 Feb 5;7(2):191752. doi: 10.1098/rsos.191752 (PMC7062053; doi:10.1098/rsos.191752)

Supporting Information Figure S1

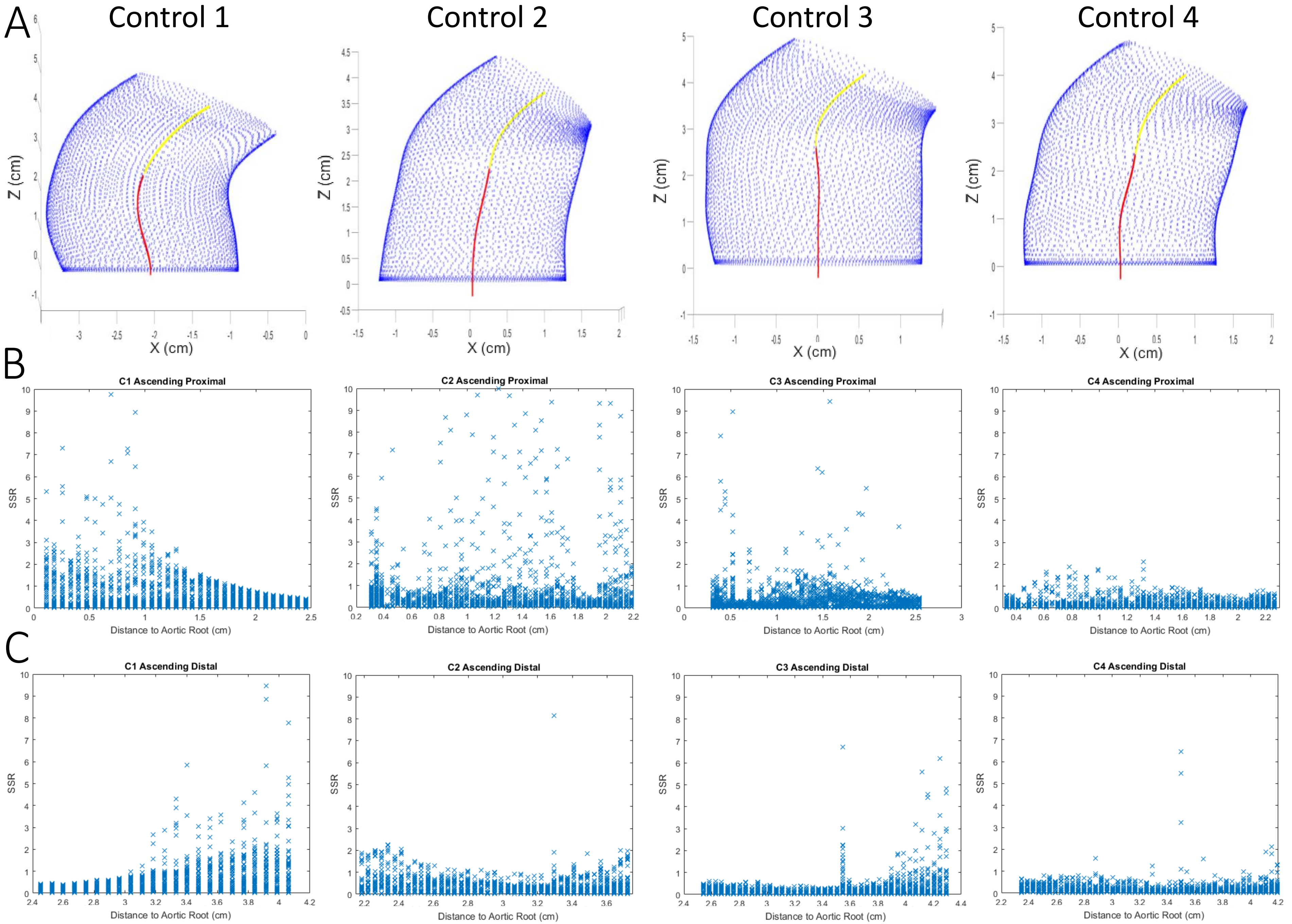

Supporting Information Figure S2

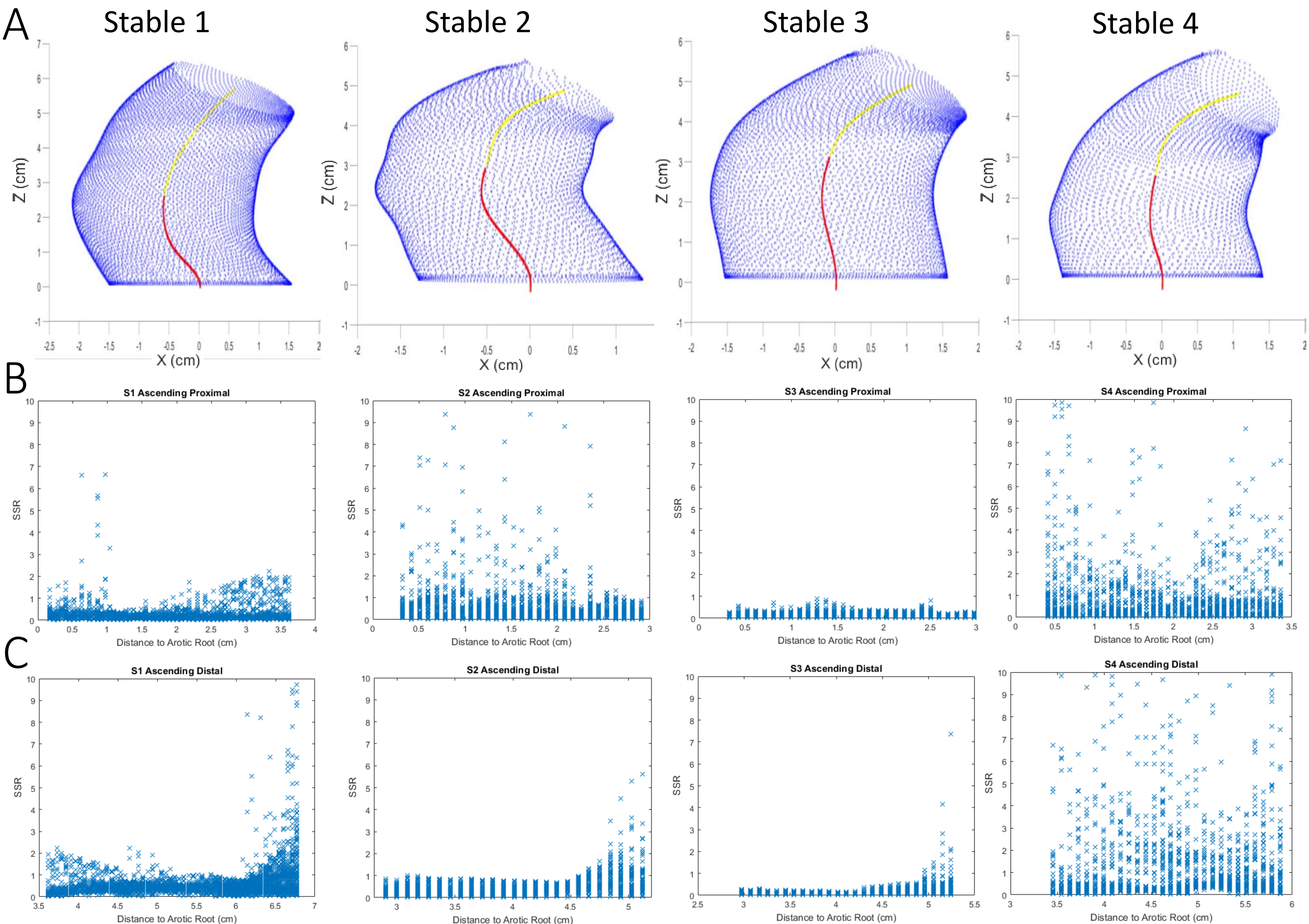

Supporting Information Figure S3

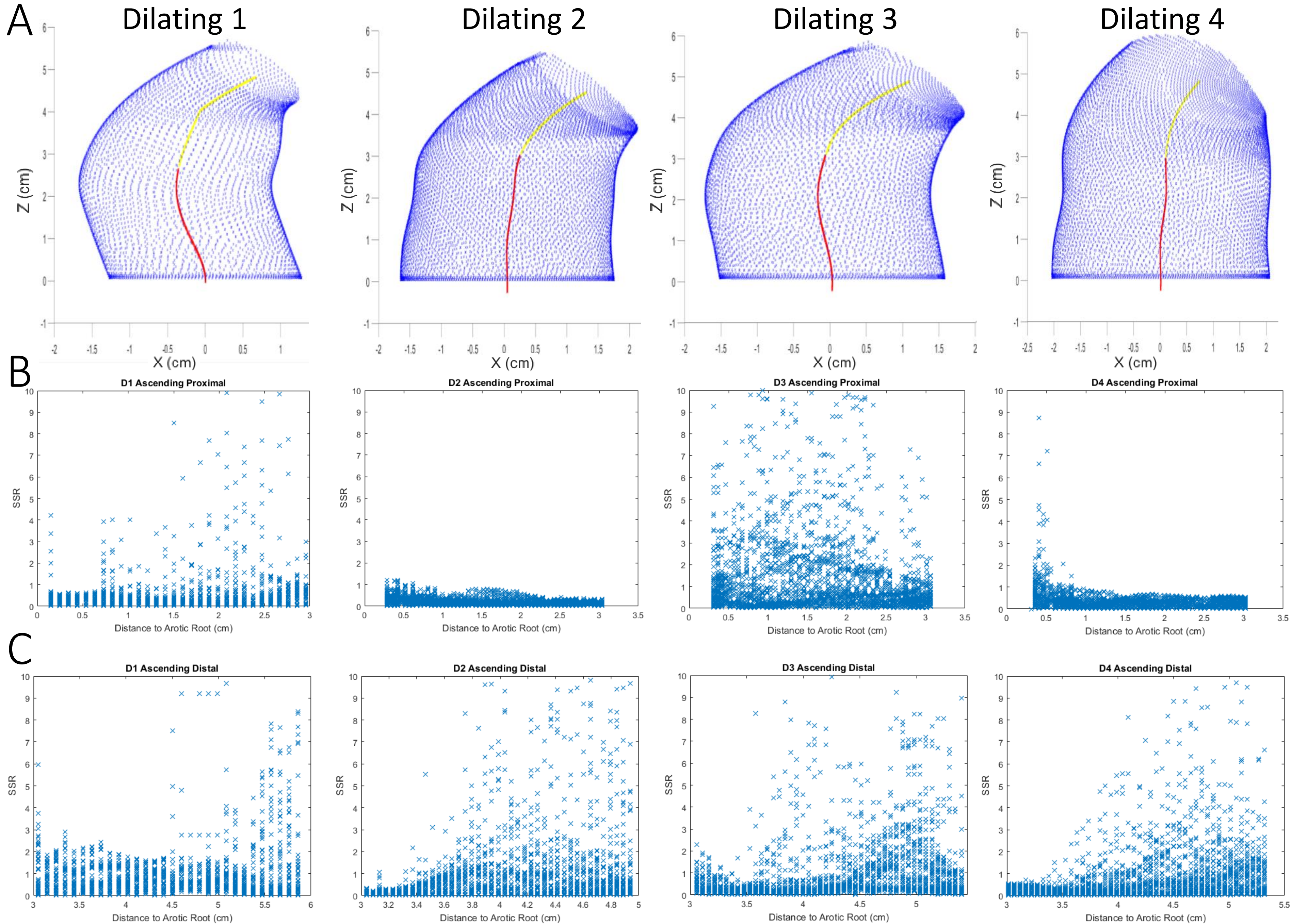

Supplement: Supplementary figures [file rsos191752supp1.pdf]
